# Impact of Introducing PROMPT on Permanent Brachial Plexus Injury and Tears III°/IV° in Shoulder Dystocia: The Hanover Cohort Study

**DOI:** 10.1155/2024/8712553

**Published:** 2024-02-03

**Authors:** Spyridon Papageorgiou, Lars Brodowski, Halina Huppertz, Bettina Bohnhorst, Markus Flentje, Constantin von Kaisenberg

**Affiliations:** ^1^Department of Obstetrics, Gynaecology and Reproductive Medicine, Hannover Medical School, Carl Neuberg Str. 1, Hannover 30625, Germany; ^2^Department of Obstetrics and Gynaecology, University Witten-Herdecke, Marien Hospital Witten, Marienplatz 2, Witten 58452, Germany; ^3^Department of Neonatology, Hannover Medical School, Carl Neuberg Str. 1, Hannover 30625, Germany; ^4^Department of Anaesthesiology, Hannover Medical School, Carl Neuberg Str. 1, Hannover 30625, Germany

## Abstract

**Objective:**

To test the hypothesis that PROMPT reduces permanent brachial plexus palsy and perineal tears.

**Design:**

A prospective/retrospective cohort study. *Setting*. Hanover Medical School, Germany. *Population/Sample*. A self-selected population.

**Methods:**

The training period is from November 9^th^, 2017, until December 31^st^, 2019; control: January 1^st^, 2004, until November 8^th^, 2017. *Main Outcome Measures*. Shoulder dystocia, nonpermanent and permanent brachial plexus injuries (BPIs), perineal tears III°/IV°, manual manoeuvres, and asphyxia.

**Results:**

There was a total of 22,640 births, and shoulder dystocia increased from 48/18,031 (0.27%) to 23/4,609 (0.50%) ((*p*=0.017), OR: 1.88, 95% CI: (1.14; 3.09)), whereas BPIs decreased from 7/48 (14.6%) to 1/23 (4.3%) (*p*=0.261). There was 1/7 (14.2%) of permanent BPI before and 0/1 (0%) case after. Perinatal asphyxia increased from 3/48 (6.3%) to 4/23 (17.4%) (*p*=0.23). However, adverse outcomes after one year were zero. McRoberts' manoeuvre increased from 37/48 (77.1%) to 23/23 (100%) ((*p*=0.013), OR: 1.62, 95% CI: (1.33; 1.98)), and internal rotation manoeuvres and manual extraction of the posterior arm from 6/48 (12.5%) to 5/23 (21.7%) (*p*=0.319). Episiotomies decreased from 5,267/18,031 (29.2%) to 836/4,609 (18.1%) ((*p* < 0.001), OR: 0.54, 95% CI: (0.49, 0.58)), whereas perineal tears III°/IV° associated with shoulder dystocia increased from 1/48 (2.1%) to 1/23 (4.8%) (*p*=0.546). Vaginal operative deliveries remained constant (6.5% vs. 7%).

**Conclusions:**

PROMPT significantly improves the management of shoulder dystocia and decreases permanent brachial plexus injuries but not perineal tears III°/IV°.

## 1. Introduction

PROMPT (Practical Obstetric Multiprofessional Training) is a trial-based multiprofessional training concept of rare obstetric complications focusing on teamwork and communication, set up in Bristol, UK, in 2000. The effectiveness of the training has been demonstrated in many trials (https://www.promptnz.org/evidence-of-effectiveness). In 2017, PROMPT was introduced at the Hannover Medical School in Germany.

Shoulder dystocia is a rare but severe obstetrical emergency, leading to severe complications for the newborn, such as brachial plexus injury (BPI), brain damage due to hypoxia, and fetal death. It may also seriously harm the mother. The actual incidence of shoulder dystocia is thought to be between 2-3% worldwide [1]. Risk factors have been identified, such as fetal macrosomia, type I diabetes or gestational diabetes, shoulder dystocia in a previous pregnancy, labour induction, epidural anaesthesia, vaginal operative delivery, and very short and prolonged second stage of labour [2]. However, these risk factors have a low predictive value, and many cases of shoulder dystocia are not associated with any of these parameters. Brachial plexus injury is the most common complication of shoulder dystocia, with an incidence of 0.5–4.0 per 1000 cases [3]. It has been shown in many studies that the implementation of training can reduce the incidence of BPIs [4]. A retrospective cohort study by Draycott et al. found that practical training (PROMPT) led to a significant reduction of BPIs from 7.4% to 1.3% (*p* < 0.01). After suitable training, they could even reduce permanent BPIs to 0% [5].

Perineal tears III°/IV° have a prevalence rate from 0.6% to 8% [6]. Shoulder dystocia and internal manoeuvres were associated with a three-fold increase in the risk of OASIS (obstetric anal sphincter injuries) [7]. However, there are no studies investigating if training for managing shoulder dystocia can reduce perineal tears III°/IV° for the mother.

This is a prospective and retrospective observational cohort study to test the hypothesis that PROMPT minimizes the mother's permanent brachial plexus injury and birth injuries after 2 and 1/2 years of training at the Hannover Medical School.

## 2. Methods

In this prospective observational cohort study, we compared the frequencies of shoulder dystocia, permanent brachial plexus injury, and perineal tears III°/IV° following the introduction of PROMPT at the Hanover Medical School in 2017 with retrospective data from 2004.

All professional groups involved in labour and childbirth (40 obstetricians, 42 midwives, 4 neonatologists, 4 paediatric nurses, 7 anaesthetists, 6 anaesthesia nurses, and 6 theatre nurses) participated in the training.

The impact of training following its introduction was assessed by comparing complication rates before and after the implementation. All infants born during 16 years, from January 1^st^, 2004, until December 31^st^, 2019, were identified using a computerized fetal database (Viewpoint V.5 and V.6). In addition, all birth books from 2004 until 2019 were examined and checked by hand to identify further cases. All cases of neonates with suspected plexus injury following shoulder dystocia were followed up at the age of one year, and the outcomes were recorded. The study compared the period “before the training” (group 1) and “after the training” (group 2) regarding the number of shoulder dystocia cases, brachial plexus injuries, asphyxia, and adverse outcomes, as well as the frequency of the manoeuvres performed. In particular, the numbers of perineal tears III°/IV°, episiotomies, and vaginal operative deliveries were compared.

### 2.1. Statistical Methods

The statistical evaluation mainly employed basic descriptive statistics. For qualitative variables, absolute and percent frequencies were given. The two study groups (before PROMPT and after the initiation of the PROMPT) were contrasted using contingency tables and tested for significant differences using the chi-square test or, if the expected frequencies proved too low, Fisher's exact test was applied. Odds ratios with 95% CI were calculated in case of significant differences. Relative risk reduction was also calculated for all qualitative parameters. Quantitative variables were presented as the mean with standard deviation or the median with range. They were tested for normal distribution using the Kolmogorov–Smirnov test. In case of significant deviations from the normal distribution, both groups were compared using the Mann–Whitney *U* test; otherwise, the independent samples *t*-test was used.

Statistical tests were performed two sided at a significance level of 5%. Due to the descriptive nature of the present analysis, no alpha adjustment for multiple testing was applied, and the results were interpreted accordingly.

Statistical analyses were undertaken using IBM SPSS Statistics 26 (SPSS Inc., an IBM Company, Chicago, IL).

Ethical approval was obtained from the Hannover Medical School Ethical Committee (Nr. 8268 BO K 2019, 16^th^ January 2019).

## 3. Results

There was total of 22.640 vaginal deliveries, 18.031 in group 1 and 4.609 in group 2, respectively. The main findings are an increase of cases diagnosed with shoulder dystocia, an increase of release manoeuvres for the anterior shoulder, and in particular a reduction of permanent brachial plexus injuries. Also, episiotomies were performed less frequently but perineal tears III°/IV° increased.

### 3.1. Shoulder Dystocia

The main findings were an increase in the diagnosis of shoulder dystocia (48/18.031 (0.27%)–23/4.609 (0.50%) (*p*=0.017), OR: 1.88, 95% CI: (1.14; 3.09)) and of McRoberts' manoeuvres (37/48 (77.1%)–23/23 (100%) (*p*=0.013), OR: 1.62, 95% CI: (1.33; 1.98)), whereas, simultaneously, the numbers of permanent brachial plexus injury (BPI) decreased (1/7 (14.2%) vs. 0/1 (0%)). Also, the use of internal rotation manoeuvres and manual extraction of arms was improved (6/48 (12.5%)–5/23 (21.7%) (*p*=0.319)).

The diagnosis of shoulder dystocia before and after the training almost doubled (48/18.031 (0.27%)–23/4.609 (0.50%) (*p*=0.017), OR: 1.88, 95% CI: (1.14; 3.09)). BPIs decreased from 14.6% to 1/23 (4.3%) (*p*=0.261). There was 1/7 (14.2%) of permanent BPI (group 1) and 0/1 (0%) cases (group 2), respectively.

Beside BPI, the second most severe adverse outcome in shoulder dystocia was asphyxia, which had a long-term negative impact on the newborn and the family. Here, the results were unexpected as birth asphyxia increased substantially from 3/48 (6.3%) to 4/23 (17.4%) (*p*=0.23); there were, however, no adverse effects of perinatal asphyxia at one year of age in either of the groups.

The most important finding for manoeuvres was a statistically significant increase in performing McRoberts (37/48 (77.1%)–23/23 (100%) (*p* = 0.013), OR: 1.62, 95% CI: (1.33; 1.98)) and a clinical increase in suprapubic pressure (13/48 (27.1%)–7/23 (30.4%) (*p* = 0.784)). Also, internal rotational manoeuvres, including manual extraction of the arm, were more frequently applied (from 6/48 (12.5%) to 5/23 (21.7%) (*p* = 0.319)), which was, however, not statistically significant (Tables 1 and 2).

The descriptive analysis showed no significant differences between the groups regarding gestational age, gravida, para, and birthweight, indicating similarity between both populations of infants and mothers.

The outcome parameters 5′ APGAR score, umbilical artery pH, and base excess were similar in both groups. The slight increase in maternal blood loss recordings (group 2) may indicate either an increased blood loss or an improved estimation and documentation. However, there were no statistically significant differences between the groups (Table 3).

In detail, we observed an increase in the cases with 5′ APGAR score <7 from 4/48 (8.3%) to 4/23 (17.4%). An increase of umbilical artery pH ≤7.10 from 7/47 (14.9%) to 4/23 (17.4%), an increase of the base excess ≤−12 from 6/47 (12.8%) to 4/23 (17.4%), as well as an increase in blood loss ≥500 ml from 2/48 (4.2%) to 3/23 (13%) were noticed. The percentage of nulliparous women with shoulder dystocia increased from 19/48 (39.6%) to 12/23 (52.2%). Also, the proportion of women in post-term pregnancies (≥40^+0^ wks. of gestation) increased from 21/48 (43.8%) to 12/23 (52.2%). However, the mean birthweight was slightly reduced from 4,062.5 g to 3,910 g (3.8% RRR).

### 3.2. Perineal Tears III°/IV°

The main findings following the training are a substantial decrease in episiotomies (5,267/18,031 (29.2%)–836/4,609 (18.1%) (*p* < 0.001), OR: 0.54, 95% CI: (0.49, 0.58)). In contrast, the total number of perineal tears III°/IV° increased from 92/17.939 (0.5%) to 39/4.570 (0.85%) ((*p*=0.007) OR: 1.66, 95% CI: (1.14; 2.42)). However, the number of cases with perineal tears III°/IV° following shoulder dystocia was not significantly different; there was a slight increase from 1/48 (2.1%) to 1/23 (4.3%) (*p*=0.546).

The percentage of vaginal operative deliveries remained relatively constant (1178/18031 (6.5%) and 324/4609 (7%)) between the groups.

The number of perineal tears III°/IV° following a spontaneous delivery, excluding vaginal operative deliveries, increased from 71/16,853 (0.42%) to 26/4,285 (0.60%). Again, this was not statistically significant (*p*=0.1).

In the subgroup of total vaginal operative deliveries, we observed a statistically significant increase in the frequency of perineal tears III°/IV° from 21/1.178 (1.78%) to 13/324 (4.0%) ((*p*=0.017), OR: 2.3, 95% CI: (1.14; 4.65)) (Table 4).

The descriptive analysis of both groups showed no demographic differences between the populations of infants and mothers in the groups.

We applied the national policy and did not apply routine episiotomy in every case of operative vaginal delivery. As a result, while the number of vaginal operative deliveries remained constant, episiotomies decreased substantially and perineal tears III°/IV° increased (Table 5).

## 4. Discussion

### 4.1. Main Findings

After 2 and 1/2 years of PROMPT, the total BPIs were reduced from 14.6% to 4.3% and permanent BPIs from 14.28% to 0%. Simultaneously, McRoberts' manoeuvre increased from 77.1% to 100%. During this period, episiotomies went from 29.1% to 18.1% but total perineal tears III°/IV° increased from 0.5% to 0.85%. Base excess and blood loss went up slightly (n.s.).

PROMPT brakes with the dogma that is rare but severe obstetrics complications cannot be trained. Specific manoeuvres such as McRoberts, suprapubic pressure, internal rotation, delivery, and the development of the posterior arm are well established to resolve the emergencies, and positive effects have been demonstrated [5, 8]. Draycott et al. [9] have shown that PROMPT resulted in similar rates of shoulder dystocia before and after the training (2.04% and 2.00%, respectively), but the initiation of release manoeuvres doubled; in particular, McRoberts' manoeuvre (from 29.3% to 87.4%, respectively). Our results are in line with Draycott et al., as McRoberts' manoeuvre increased from 77% to 100%.

The study of Draycott et al. [9] demonstrated a reduction of brachial plexus injury at birth from 10% to 2.3%. Weiner et al. [10] also showed a similar reduction from 10.7% to 0% in five years. Again, our study is in line with the results published in the literature following PROMPT, as the total brachial plexus injury at birth went from 14.6% to 4.3% and the permanent BPI from 14.2 to 0%, albeit in small numbers. These results show a very promising impact of the training and are the study's most important findings. Ongoing annual training is, however, mandatory to make these results sustainable [11, 12].

Perineal tears III°/IV° can cause serious short- and long-term harm for the mothers, including fecal incontinence [13] and sexual dysfunction [14]. Therefore, their prevention is critically important. The use of episiotomy is a controversial issue. Some observational studies have shown that the use of episiotomy for vaginal births [15] and vaginal operative deliveries (vacuum and forceps) [16] is associated with a risk reduction. However, other evidence has failed to show a protective impact of the episiotomy [6] or an increase of third- and fourth-degree tears associated with episiotomies [17]. In particular, the study of Shmueli et al. [18] has shown a 2.26-fold increased incidence of perineal tears III°/IV° following an episiotomy after a spontaneous delivery but no differences after vaginal operative deliveries. Our analysis showed an increase of perineal tears III°/IV° from 0.51% to 0.85% following the PROMPT with a simultaneous decrease of the use of episiotomy from 29.2% to 18.1%. This may indicate a potential protective effect of episiotomies for the risk of perineal tears III°/IV°, particularly in vaginal operative deliveries. As observed, the incidence of vaginal operative deliveries resulting in third- and fourth-degree tears increased from 1.8% to 4% after the training. However, their percentage remained unchanged in both groups at around 7%, while the mean fetal weight increased slightly from 3,517 g to 3,604 g. This finding may be the result of the reduced use of episiotomy. However, other factors, such as an increase in diabetes [19], maternal weight [20, 21], and the improvement in documentation following the PROMPT, may have a role.

### 4.2. Strengths and Limitations

The main strength of our research was that it was conducted in a large obstetric centre with around 3000 births annually. A search of the database and all birth hard-copy recordings from 2004 provided us with comprehensive data. Also, the training concept included all professional groups involved in labour and childbirth (obstetricians, midwives, neonatologists, paediatric nurses, anaesthetists, anaesthesia nurses, and theatre nurses). Therefore, the whole team worked together using the same management algorithms. The training was repeated four times annually.

Several limitations have to be mentioned. First, the participation level of staff was low. Over the 2 and 1/2-year period, we observed a total participation rate of only 35.6%. However, among the core staff involved, the participation rates were significantly higher, with obstetricians achieving a rate of 50–60% and midwives of 92–100%.

Second, there was a low total number of rare obstetrical complications such as shoulder dystocia cases (71/22,640) and III°/IV° perineal tear cases (131/22,640), limiting the conclusions that can be drawn from this cohort.

### 4.3. Interpretation

The authentical use of a scientifically written and effective training concept, the adaptation of the training materials into the German language taking into account German guidelines and drugs, the use of high fidelity simulators, and the implementation of the PROMPT concept involving teams of midwives, obstetricians, paediatricians, and anaesthesiologists on a labour ward with more than 3,000 deliveries per annum and with a wide variety of high-risk pregnancies have resulted in significant improvements after only two and a half years. This approach can reduce permanent brachial plexus injury in shoulder dystocia to zero.

The findings of the PROMPT introduction at a German-speaking university hospital show substantial benefits. Albeit in a relatively small number of cases, the cohort represents the clinical reality in a German setting rather well.

## 5. Conclusion

PROMPT significantly improves the management of shoulder dystocia and decreases permanent brachial plexus injuries but not perineal tears III°/IV°.

## Figures and Tables

**Table 1:** Shoulder dystocia cases and manoeuvres applied (mothers).

	Group		*p* value
	1	2	
Shoulder dystocia total cases	48/18031 (0.27%)	23/4609 (0.50%)	**0.017**
McRoberts' manoeuvre	37/48 (77.1%)	23/23 (100%)	**0.013**
Suprapubic pressure	13/48 (27.1%)	7/23 (30.4%)	0.784
Internal rotation and manual arm extraction	6/48 (12.5%)	5/23 (21.7%)	0.319

Significance at *p* < 0.05. The bold highlight the “statistically significant” findings.

**Table 2:** Brachial plexus injury, asphyxia, and adverse outcomes (infants).

	Group		*p* value
	1	2	
Total brachial plexus injury (BPI)	7/48 (14.6%)	1/23 (4.3%)	0.261
Permanent BPI	1/7 (14.2%)	0/1 (0%)	n.a.^*∗*^
Total cases complicated with asphyxia	3/48 (6.3%)	4/23 (17.4%)	0.23
Adverse outcome after one year	0/3 (0%)	0/4 (0%)	n.a.^*∗*^

Significance at *p* < 0.05. n.a.^*∗*^: the statistical significance calculation is unavailable for these groups (0 cases in group 2).

**Table 3:** Laboratory parameters and demographic characteristics (infants and mothers).

Groups	Categories	*N*	Mean	Min	Max	Centiles		
						25^th^	50^th^ median	75^th^
1	Neonatal 5′ APGAR score	48	8.75	2	10	8	9	10
	Neonatal umbilical artery pH	47	7.20	6.99	7.4	7.16	7.2	7.29
	Neonatal base excess	47	−7.74	−19.1	1.7	−10.5	−6.7	−5.3
	Gestational age (weeks)	48	39.6	37	41	39	40	40
	Gestational age at delivery (days)	48	279.48	261	290	276	280	285.75
	Gravida	48	2.56	1	7	1	2	3
	Para	48	2.02	1	6	1	2	2
	Neonatal birthweight	48	3,945.21	2,785	4,885	3,652.5	4,062.5	4,303.75
	Maternal blood loss	48	**297.92**	150	2000	200	**225**	300

2	Neonatal 5′ APGAR score	23	8.17	4	10	8	9	9
	Neonatal umbilical artery pH	23	7.21	7.03	7.47	7.15	7.2	7.29
	Neonatal base excess	23	−7.78	−26	−0.7	−10.3	−7.4	−3.9
	Gestational age (weeks)	23	39.57	33	41	39	40	41
	Gestational age at delivery (days)	23	279.39	231	291	277	281	289
	Gravida	23	2.13	1	8	1	2	2
	Para	23	1.78	1	6	1	1	2
	Neonatal birthweight	23	3,941.74	2,230	4,830	3,605	3,910	4,350
	Maternal blood loss	23	**375.22**	150	1300	200	**300**	350

The bold values highlight the most important findings.

**Table 4:** Perineal tears III°/IV°, episiotomies, and vaginal operative deliveries (mothers).

	Group		*p* value
	1	2	
Perineal tears III°/IV° (general population)	92/18,031 (0.5%)	39/4,609 (0.85%)	**0.007**
Perineal tears (III°/IV°)/shoulder dystocia	1/48 (2.1%)	1/23 (4.3%)	0.5
Episiotomies	5,267/18,031 (29.2%)	836/4,609 (18.1%)	**<0.001**
Vaginal operative deliveries	1,178/18,031 (6.5%)	324/4,609 (7.0%)	0.23
Perineal tears III°/IV°, **e**xcluding vaginal operative deliveries	71/16,853 (0.42%)	26/4,285 (0.6%)	0.1
Perineal tears III°/IV°, following vaginal operative deliveries	21/1,178 (1.8%)	13/324 (4.0%)	**0.017**

Significance at *p* < 0.05. The bold values highlight the “statistically significant” findings.

**Table 5:** Demographic characteristics between group 1 and group 2 (infants and mothers).

Group		*N*	Mean	Min	Max	Centiles		
						25^th^	50^th^ median	75^th^
1	Length of pregnancy (d)	92	277.8	236	293	273	280	284
	Maternal age (y)	92	30,61	20	47	27	30,5	34
	Gravida	92	1.52	1	5	1	1	2
	Para	92	1.18	1	3	1	1	1
	Neonatal birthweight	92	3,517.7	2,105	4,530	3,252.5	3,540	3,841.2
	Neonatal head circumference	92	35.06	30	38	34	35	36

2	Length of pregnancy (d)	39	279.1	249	293	273	281	287
	Maternal age (y)	39	31,56	25	41	29	31	33,5
	Gravida	39	1.28	1	3	1	1	1
	Para	39	1.13	1	2	1	1	1
	Neonatal birthweight	39	3,604.1	2,950	4,405	3,310	3,660	3,810
	Neonatal head circumference	39	35.19	33	37.5	34	35	36

## Data Availability

The data used to support the findings of this study are included within the article.

## References

[1] Menticoglou S. (2018). Shoulder dystocia: incidence, mechanisms, and management strategies. *International Journal of Women’s Health*.

[2] Wagner R. K., Nielsen P. E., Gonik B. (1999). Shoulder dystocia. *Obstetrics & Gynecology Clinics of North America*.

[3] Lalka A., Gralla J., Sibbel S. E. (2020). Brachial plexus birth injury: epidemiology and birth weight impact on risk factors. *Journal of Pediatric Orthopaedics*.

[4] Dahlberg J., Nelson M., Dahlgren M. A., Blomberg M. (2018). Ten years of simulation-based shoulder dystocia training- impact on obstetric outcome, clinical management, staff confidence, and the pedagogical practice- a time series study. *BMC Pregnancy and Childbirth*.

[5] Crofts J. F., Lenguerrand E., Bentham G. L. (2016). Prevention of brachial plexus injury- 12 years of shoulder dystocia training: an interrupted time-series study. *BJOG: An International Journal of Obstetrics and Gynaecology*.

[6] Frigerio M., Manodoro S., Bernasconi D. P., Verri D., Milani R., Vergani P. (2018). Incidence and risk factors of third- and fourth-degree perineal tears in a single Italian scenario. *European Journal of Obstetrics & Gynecology and Reproductive Biology*.

[7] Gauthaman N., Walters S., Tribe I. A., Goldsmith L., Doumouchtsis S. K. (2016). Shoulder dystocia and associated manoeuvres as risk factors for perineal trauma. *International Urogynecology Journal*.

[8] Davis D. D., Roshan A., Canela C. D., Varacallo M. (2022). *Shoulder Dystocia*.

[9] Draycott T. J., Crofts J. F., Ash J. P., Wilson L. V. (2008). Improving neonatal outcome through practical shoulder dystocia training. *Obstetrics & Gynecology*.

[10] Weiner C., Samuelson L., Collins L., Satterwhite C. (2014). 61: 5-year experience with PROMP (PRactical Obstetric Multidisciplinary Training) reveals sustained and progressive improvements in obstetric outcomes at a US hospital. *American Journal of Obstetrics and Gynecology*.

[11] Cornthwaite K., Alvarez M., Siassakos D. (2015). Team training for safer birth. *Best Practice & Research Clinical Obstetrics & Gynaecology*.

[12] Menticoglou S. (2018). Shoulder dystocia: incidence, mechanisms, and management strategies. *International Journal of Women’s Health*.

[13] Faltin D. L., Sangalli M. R., Roche B., Floris L., Boulvain M., Weil A. (2001). Does a second delivery increase the risk of anal incontinence?. *BJOG: An International Journal of Obstetrics and Gynaecology*.

[14] Signorello L. B., Harlow B. L., Chekos A. K., Repke J. T. (2001). Postpartum sexual functioning and its relationship to perineal trauma: a retrospective cohort study of primiparous women. *American Journal of Obstetrics and Gynecology*.

[15] Clemons J. L., Towers G. D., McClure G. B., O’Boyle A. L. (2005). Decreased anal sphincter lacerations associated with restrictive episiotomy use. *American Journal of Obstetrics and Gynecology*.

[16] Lund N. S., Persson L. K. G., Jangö H., Gommesen D., Westergaard H. B. (2016). Episiotomy in vacuum-assisted delivery affects the risk of obstetric anal sphincter injury: a systematic review and meta-analysis. *European Journal of Obstetrics & Gynecology and Reproductive Biology*.

[17] Dandolu V., Chatwani A., Harmanli O., Floro C., Gaughan J. P., Hernandez E. (2005). Risk factors for obstetrical anal sphincter lacerations. *International Urogynecology Journal*.

[18] Shmueli A., Gabbay Benziv R., Hiersch L. (2017). Episiotomy- risk factors and outcomes. *The Journal of Maternal-Fetal & Neonatal Medicine*.

[19] Reicher Y., Weintraub A. Y., Baumfeld Y., Dym L., Amit K., Eshkoli T. (2022). Diabetes Mellitus in pregnancy-a risk factor for perineal injuries, independent of neonatal macrosomia. *American Journal of Obstetrics and Gynecology*.

[20] Garretto D., Lin B. B., Syn H. L. (2016). Obesity may Be protective against severe perineal lacerations. *Journal of Obesity*.

[21] Durnea C. M., Jaffery A. E., Nivedita Gauthaman S. K. D. (2018). Effect of body mass index on the incidence of perineal trauma. *International Journal of Gynecology & Obstetrics*.

[22] Papageorgiou S., Brodowski L., Lewinski H., Bohnhorst B., Kaisenberg C. V. (2023). Impact of introducting PROMPT on permanent brachial plexus injury & tears° III/IV in shoulder dystocia: the HANOVER cohort study. https://www.authorea.com/users/653972/articles/660645-impact-of-introducing-prompt-on-permanent-brachial-plexus-injury-tears-iii-iv-in-shoulder-dystocia-the-hanover-cohort-study.

